# Whole‐genome resequencing‐based QTL‐seq identified *AhTc1* gene encoding a R2R3‐MYB transcription factor controlling peanut purple testa colour

**DOI:** 10.1111/pbi.13175

**Published:** 2019-06-12

**Authors:** Yuhan Zhao, Junjie Ma, Ming Li, Li Deng, Guanghui Li, Han Xia, Shuzhen Zhao, Lei Hou, Pengcheng Li, Changle Ma, Mei Yuan, Li Ren, Jianzhong Gu, Baozhu Guo, Chuanzhi Zhao, Xingjun Wang

**Affiliations:** ^1^ Biotechnology Research Center Shandong Academy of Agricultural Sciences Shandong Provincial Key Laboratory of Crop Genetic Improvement Ecology and Physiology Jinan China; ^2^ College of Life Sciences Shandong Normal University Jinan China; ^3^ College of Life Sciences Shandong University Jinan China; ^4^ Kaifeng Academy of Agriculture and Forestry Kaifeng China; ^5^ Shandong Peanut Research Institute Shandong, Qingdao China; ^6^ Crop Protection and Management Research Unit USDA‐Agricultural Research Service Tifton GA USA; ^7^ Department of Plant Pathology University of Georgia Tifton GA USA

**Keywords:** peanut, testa colour, *AhTc1*, QTL‐seq, BSA, antioxidant

## Abstract

Peanut (*Arachis hypogaea*. L) is an important oil crop worldwide. The common testa colours of peanut varieties are pink or red. But the peanut varieties with dark purple testa have been focused in recent years due to the potential high levels of anthocyanin, an added nutritional value of antioxidant. However, the genetic mechanism regulating testa colour of peanut is unknown. In this study, we found that the purple testa was decided by the female parent and controlled by a single major gene named *AhTc1*. To identify the candidate gene controlling peanut purple testa, whole‐genome resequencing‐based approach (QTL‐seq) was applied, and a total of 260.9 Gb of data were generated from the parental and bulked lines. SNP index analysis indicated that *AhTc1* located in a 4.7 Mb region in chromosome A10, which was confirmed by bulked segregant RNA sequencing (BSR) analysis in three segregation populations derived from the crosses between pink and purple testa varieties. Allele‐specific markers were developed and demonstrated that the marker *pTesta1089* was closely linked with purple testa. Further, *AhTc1* encoding a R2R3‐MYB gene was positional cloned. The expression of *AhTc1* was significantly up‐regulated in the purple testa parent YH29. Overexpression of *AhTc1* in transgenic tobacco plants led to purple colour of leaves, flowers, pods and seeds. In conclusion, *AhTc1*, encoding a R2R3‐MYB transcription factor and conferring peanut purple testa, was identified, which will be useful for peanut molecular breeding selection for cultivars with purple testa colour for potential increased nutritional value to consumers.

## Introduction

Peanut (or groundnut), widely cultivated in more than 100 countries, with the annual production of 43.98 million tons, is one of the most important oil crops in the world (FAOSTAT 2016, www.faostat.fao.org). Peanut seeds are rich in oil (40%–60%), protein (20%–40%), carbohydrate (10%–20%) and many other nutritional values, such as vitamin B1, B3, B9 and E, biotin, resveratrol, isoflavones and phytic acid (Pandey *et al*., 2012; Zhao *et al*., 2012). The testa colours of most peanut varieties are with pink or red. Recently, the peanut varieties with purple testa have attracted increasing attention as a potential source of nutraceuticals, owing to their higher anthocyanin content and microelements, which are beneficial to human health (Attree *et al*., 2015; Kuang *et al*., 2017). Peanut varieties with purple testa satisfy the needs of the customers. However, the commercial fine varieties with black or purple testa are scarce which limits the production of purple peanuts.

In the last decade, molecular breeding approaches such as marker‐assisted selection (MAS) have been utilized in peanut and other crops, which significantly improved the efficiency of breeding. Despite the agronomic importance of testa colour, the genetic and the molecular mechanism that controls testa colour in peanut are not clear. Besides pink and purple testa, germplasms with white, red and tan testa are also available. It is reported that the purple testa is completely dominant to white and incompletely dominant to the basic tan or pink testa colour, and purple testa of peanut was controlled by a single dominant gene (Branch, 1985, 2001). A study showed that the purple testa of peanut was controlled by an incomplete dominant major gene, which linked with SSR marker *PM93* (Hong *et al*., 2007). Sequence alignment showed that SSR marker *PM93* was same as *Seq4H11*, located at 6.664 cM of linkage group TA10 in the integrated consensus map of cultivated peanut and wild relatives (Shirasawa *et al*., 2013).

Cultivated peanut (*Arachis hypogaea*. L) is an allotetraploid (AABB, 2*n* = 4× = 40). The large size of the genome (2800 Mb), the ploidy level and high content of repetitive DNA (Dhillon *et al*., 1980) have obstructed genetic and genomic studies in peanut, and make positional cloning of a gene very difficult. During the past 5 years, significant progress has been made in peanut genomic sequencing and QTL mapping. In 2016, the genome sequences of two diploid wild ancestors of cultivated peanut, *Arachis duranensis* and *Arachis ipaensis*, were determined (Bertioli *et al*., 2016; Chen *et al*., 2016). In 2017, two groups reported the completion of genome sequences of cultivated peanut, Tifrunner (Bertioli *et al*., 2019) and Shitouqi (Zhuang *et al*., 2019). In addition, the draft sequences of *Arachis monticola*, the only allotetraploid wild peanut in the *Arachis genus,* were reported (Yin *et al*., 2018). These efforts provided new opportunities for peanut genetic analysis, gene cloning, QTL mapping and molecular marker development (Luo *et al*., 2019; Pandey *et al*., 2017a,b; Wang *et al*., 2017; Zhao *et al*., 2017).

Traditional QTL analysis has been known as time‐consuming and labour‐intensive work. In comparison, bulked segregant analysis (BSA) was first reported in 1991 and is an elegant method for rapidly identifying markers linked to any specific gene or genomic region using two bulked DNA pools (Michelmore *et al*., 1991). Recent development of next‐generation sequencing technologies (NGS) reduced the cost and shortened the cycles of sequencing, promoting the use of sequence‐based trait mapping approaches. Based on the integration of BSA, NGS and bioinformatics analysis, a series of more efficient approaches were developed, including QTL‐seq, MutMup, MutMup+ and BSR‐seq. These approaches have been successfully employed in QTL mapping and candidate gene identification in several crops (Abe *et al*., 2012; Fekih *et al*., 2013; Steuernagel *et al*., 2016; Takagi *et al*., 2013). QTL‐seq is a whole‐genome resequencing (WGRS)‐based approach, which firstly to resequence two bulked DNA of progenies (each with 20–50 individuals) showing extreme phenotypic values and then identifying the candidate region or genes by counting and comparing the index SNPs between two bulks (Takagi *et al*., 2013). It is remarkable that QTL‐seq has been successfully used in peanut in mapping QTLs of rust resistance, late leaf spot resistance, and genomic regions and candidate genes controlling shelling percentage (Clevenger *et al*., 2018; Luo *et al*., 2019; Pandey *et al*., 2017a).

In this study, the inheritance of purple testa of peanut was analysed using F_2_, F_2:3_ segregation population derived from a cross between peanut varieties with pink and purple testa. A major location controlling the purple testa was mapped using QTL‐seq method. Furthermore, a candidate gene, *AhTc1*, encoding a R2R3‐MYB transcription factor, was identified using map‐based cloning method together with transcriptome analysis and gene expression profiling. Functional studies indicated that *AhTc1* played important roles in regulating anthocyanin biosynthesis. Our results laid the foundation for breeding purple testa peanut varieties using peanut MAS program.

## Results

### Genetic analysis of purple testa in YH29

**Figure 1:**
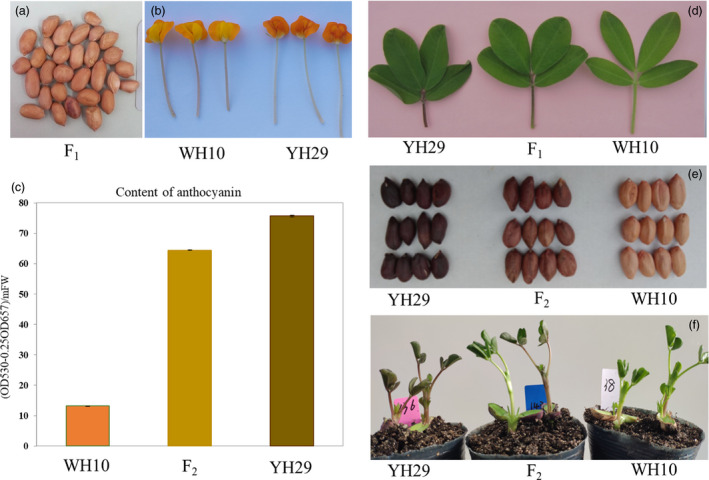
Variation in colour and content of pigments between parental lines and offsprings. (a) F_1_ seeds derived from the cross of WH10 and YH29. (b) The flower colour difference between WH10 and YH29. (c) The contents of anthocyanin in testa of parents and F_2_. (d) Leaves of parental lines and F_1_. (e) Seeds of parental lines and F_2_. (f) Seedlings of parental lines and F_2_.

YH29 and ZH9 are varieties with purple testa, while WH10, GT‐C20 and ZH8 are varieties with pink testa. In addition, among the parental lines, there was also variation in the leaflets, flower, vasculature and petiole colour where lines with darker testa also showed more purple coloration in these tissues (Figure 1). The peanut varieties with purple testa were used as male parents for crossing with the varieties with pink testa (Table 1). All F_1_ plants showed the intermediate phenotype in leaflets, vasculature, petiole and flower colour comparison with their parental lines (Figure 1). However, all F_1_ seeds were with pink testa, same as the female parents. For F_2_ and F_3_ generation, the seeds harvested from a single plant were also with same testa colour, and the testa colour was in consistence with the colour of female parents. Our results were in consistence with studies in other plants (Lambrides *et al*., 2004). So, we predicted that testa was developed by the integument of the ovule, and hence, the testa has the same genotype with the maternal plant, which lead to the segregate of testa colour in the next generation. We determined the genotype of a seed through the phenotype of seeds from KF1 (WH10♀ × YH29♂) and KF2 (GT‐C20♀ × YH29♂) populations. Statistical results corresponded to a single locus segregation ratio (Table 1). Combined with phenotypes of F_1_ plants and F_2_ seeds, we suggested that purple testa of YH29 was controlled by an incomplete dominant gene and this gene was named as *AhTc1*.

**Table 1:** Genetic analysis of purple testa colour in peanut

Name	Generation	Total plants	Purple	Light purple	Pink	Expected ratio	χ^2^	*P*‐value
KF1 WH10♀ × YH29♂	F_1_	20	0	0	20			
	F_2_	355	0	355	0			
	F_3_	355	83	178	94	1 : 2 : 1	0.231	0.05
KF2 GT‐C20♀ × YH29♂	F_1_	10	0		10			
	F_2_	107		107				
	F_3_	107	26	52	29	1 : 2 : 1	0.252	0.05
ZH ZH8♀ × ZH9♂	F_1_	8	0		8			
	F_2_	72		72				
	F_7_(RIL)	72	44		28			

### QTL‐seq predicted candidate genomic region controlling purple testa

To map *AhTc1*, KF1 population was used for QTL‐seq analysis. Two parents and two extreme bulks (purple‐pool and pink‐pool) were sequenced using Illumina HiSeq 2500 platform. In total, 116 631 629, 109 298 108, 350 931 558 and 292 791 543 read pairs (150 bp × 2) were generated from pink testa parent, purple testa parent, pink‐pool and purple‐pool, respectively. Among which, 88 487 214 (75.87%), 83 752 350 (76.63%), 266 400 406 (75.91%) and 222 527 818 (76.00%) read pairs were uniquely and confidently mapped to the reference genome, respectively (Table 2).

**Table 2:** Data generated by QTL‐seq and BSR‐seq

Populations	Samples	Raw data (Gb)	High‐quality reads (150 bp × 2)	Uniquely mapped reads (150 bp × 2)	Alignment (%)	Average depth
For QTL‐seq						
KF1	WH10	34.99	116 623 263	88 487 214	75.87	12.50
	YH29	32.79	350 904 687	266 400 406	75.91	11.71
	Purple‐pool	87.84	109 290 025	83 752 350	76.63	31.37
	Pink‐pool	105.28	292 769 375	222 527 818	76.00	37.60
For BSR‐seq						
KF1	WH10	4.34	14 462 145	11 341 989	78.43	
	YH29	4.74	15 812 681	12 285 282	77.69	
	Purple‐pool	7.93	26 442 982	20 577 921	77.82	
	Pink‐pool	8.97	29 907 000	23 786 858	79.54	
KF2	GT‐C20	3.86	12 851 990	10 415 745	81.04	
	YH29	4.74	15 812 681	12 285 282	77.69	
	Purple‐pool	8.80	29 315 586	23 489 773	80.13	
	Pink‐pool	8.30	27 654 103	21 936 469	79.32	
ZH	ZH8	3.89	12 951 240	10 319 990	79.68	
	ZH9	4.55	15 162 715	12 275 279	80.96	
	Purple‐pool	11.06	36 875 909	29 091 397	78.89	
	Pink‐pool	9.18	30 611 925	24 335 624	79.50	

**Figure 2:**
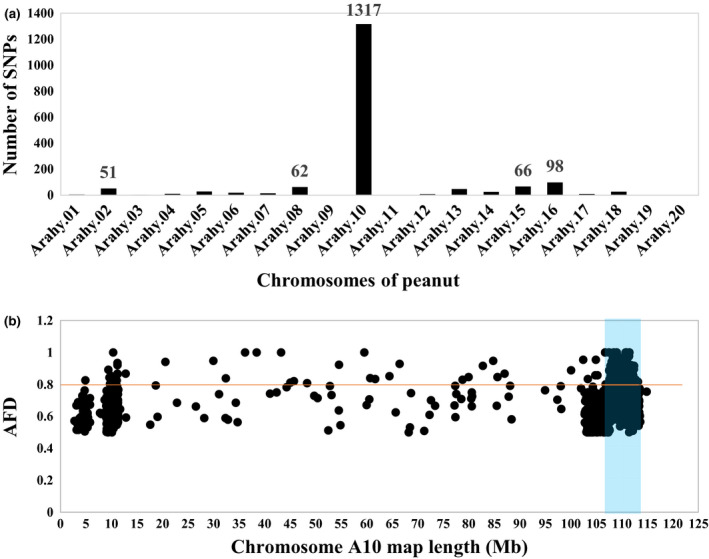
Mapping of genomic region controlling purple testa. (a) Distribution of candidate SNPs per chromosomes (AFD > 0.5, *P*‐value < 1e^−5^), (b) AFD plot of chromosome A10. The significant region identified for purple is shaded (108.0–112.7 Mb).

By comparing the two extreme bulks, a total of 640 757 high‐quality genome‐wide SNPs were called (Table S1).With a filtration criterion of allele frequency difference (AFD) >0.5 and Fisher exact test *P‐*value < 1e^−5^, a total of 1797 SNPs were putatively associated with the purple testa phenotype, and 1317 (73.29%) of them were located on chromosome A10 (Figure 2a), suggesting that the purple testa controlling gene was located on A10. SNP index analysis showed that the region on A10 from 108.0 Mb to 112.7 Mb exhibited significant unequal contributions. In this 4.7 Mb region, the average AFD value was 0.782, and 214 SNPs with AFD > 0.8, which was the highest in peanut genome, suggesting that the region might contain candidate gene controlling purple testa (Figure 2b).

### Confirmation of the candidate region using BSR‐seq in three populations

Since the phenotype variation can be observed through the colour of the leaflets between parental lines and individual of F_2_, the candidate gene could be an expressing gene. Therefore, RNA bulks for extreme phenotypes were constructed using KF1 population (F_2_), KF2 population (F_2_) and ZH (ZH8♀ × ZH9♂) RIL population. BSR sequencing generated a total 25.99, 25.69 and 28.68 Gb raw data from KF1, KF2 and ZH population, respectively (Table 2). A total of 135, 51 and 172 SNPs putatively associated with the purple testa phenotype were identified, and 71.11%, 33.34% and 65.70% of these SNPs from KF1, KF2 and ZH population were in chromosome A10, respectively (Figure S1). BSR‐seq results also showed that the SNPs were enriched in the same candidate region (Chr. A10:108.0…112.7 Mb), which confirmed the results of QTL‐seq (Figure S1).

### Development of STS markers, validation and narrowing the candidate genes

**Figure 3:**
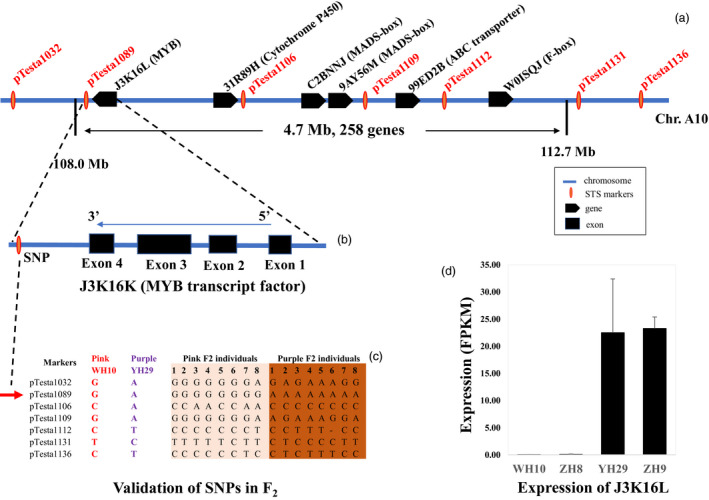
Cloning and expression analysis of *AhTc1*. (a) SNP markers and genes in candidate region of *Arachis hypogaea* chromosome A10. (b) The location and gene structure of J3K16K. (c) Genotyping results of seven SNP markers in F_2_ population. (d) The expression level of J3K16K in leaves of purple and pink testa varieties.

To narrow the candidate SNPs, 10 STS markers were developed in the candidate region on chromosome A10 range from Arahy.10:103268353 to 113621464 (Figure 3a). Seven out of these markers showed good amplification and polymorphism between parents (Table 3). Linkage of these SNPs to *AhTc1* was confirmed by testing eight pink testa and eight purple F_2_ lines. Genotyping result demonstrated that the marker *pTesta1089* (Arahy.10:108900285) was closely linked with *AhTc1* (Figure 3c). Comparative genomic analysis showed that there are 258 genes in the 4.7 Mb region (Table S2). According to functional annotation, nine candidate genes including three MYB transcription factor genes, a bHLH gene, two MADX‐box transcription factor genes, an ABC transporter gene, a F‐box transcription factor gene and a cytochrome P450 gene were highlighted (Figure 3a, Table S2). The SNP marker *pTesta1089* located in the 3′ of the MYB transcription factor (gene name: J3K16K), and the distance between them is only 2724 bp (Figure 3b). Interestingly, RNA‐seq result showed that gene (J3K16K) was highly expressed in the purple testa varieties (YH29 and ZH9) to compare with that in the pink testa varieties (WH10 and ZH8) (Figure 3d). These results implied that J3K16L was the candidate gene of *AhTc1*.

**Table 3:** Polymorphic markers linked to *AhTc1*

Primers	SNP location	AFD	Forward primer	Product length (bp)
*pTesta1032*	Arahy.10:103268353	0.65	F: GAATAGATCATCATATATTGAAA R: TTCAAACTCGTATACCCAGTAG	696
*pTesta1089*	Arahy.10:108900285	0.74	F: TTGTAAAGAGAATTAAGACGACGGA R: GCAGGTTTGTTCGCAGGA	637
*pTesta1106*	Arahy.10:110603758	0.83	F: AAAAGGGATTGGTTCGTC R: CCCTATTAAATCTACCTGAT	759
*pTesta1109*	Arahy.10:110962253	0.77	F: CATTTGTGCACTTGAATAC R: CTTTCTAATGGTCCTCAAT	791
*pTesta1112*	Arahy.10:111238108	0.82	F: TTGACAATAAAAAATGAGG R: GCCTTTAGCTCAATGAT	827
*pTesta1131*	Arahy.10:113126877	0.60	F: CAGATTTTGATAATGATTTGTGGTA R: TTTTTTATTTTACATTTATGTTTGA	765
*pTesta1136*	Arahy.10:113621464	0.57	F: ATAGTAAAAATACTGGAAC R: ATTCACGCGACTATCTCAA	652

### Function validation of the candidate gene

**Figure 4:**
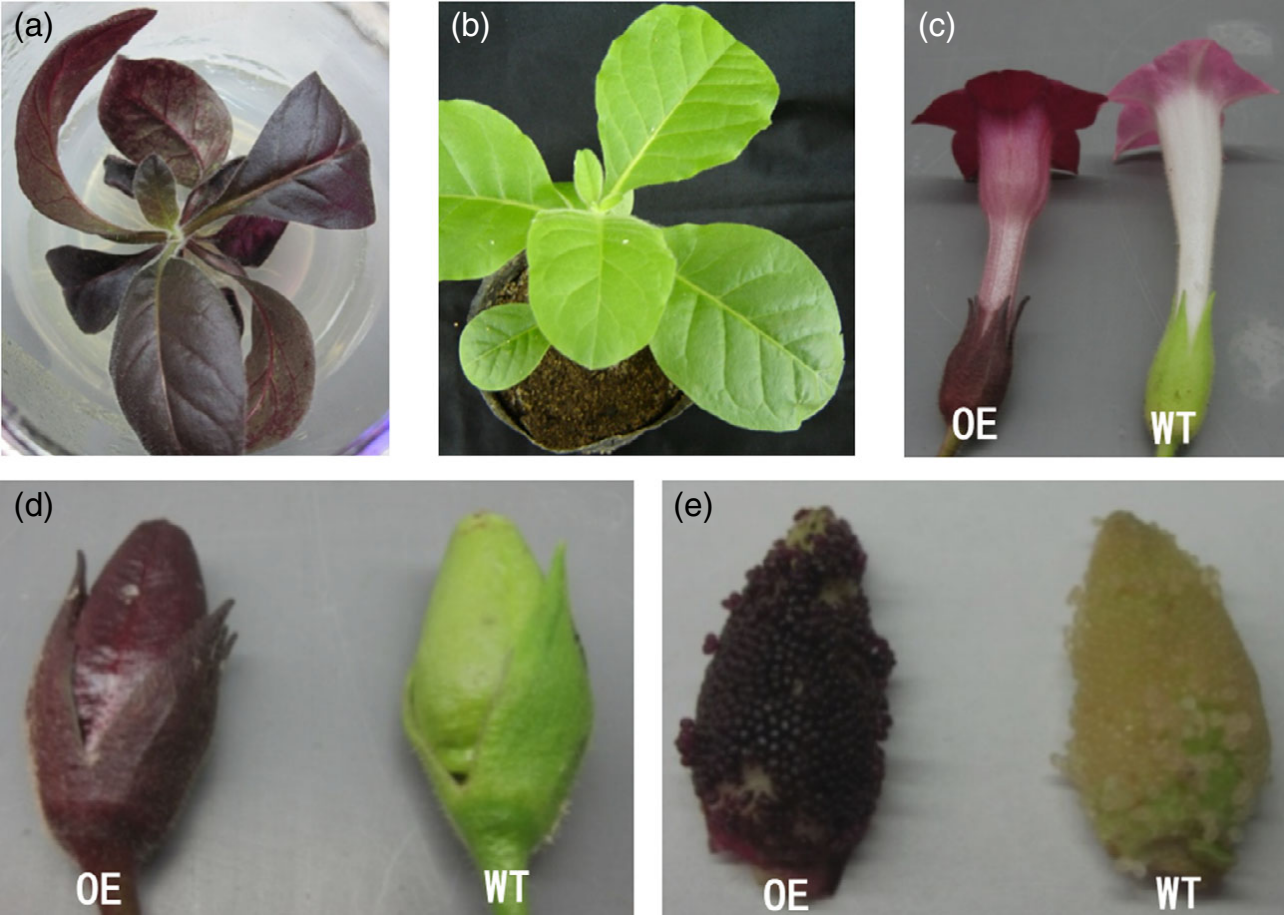
Phenotypic analysis of transgenic tobacco. (a) The transgenic seedling overexpressing J3K16K gene. (b) Nontransgenic tobacco plant. (c) Flowers of transgenic plant and wild type. (d) Fruits of transgenic plant and wild type. (e) Seeds of transgenic plant and wild type.

To confirm that the purple testa was caused by the expression of the candidate gene J3K16L, we cloned this gene (Table S3). The coding sequence of this gene, driven by CaMV 35S promoter, was introduced into the tobacco. All transgenic lines expressing J3K16L exhibited different degrees of purple colour in leaves and flowers, while the fruits and testa were all showed dark purple colour (Figure 4). In addition, the seed coats of the transgenic lines exhibited also showed more purple (Figure 4). These results confirmed that J3K16L promoted anthocyanin accumulation in transgenic tobacco plants. In peanut, the expression level of J3K16L was also positive correlated with the purple testa and the accumulation of anthocyanin (Figure 1e, Figure 3d). Taken together, our work demonstrated that J3K16L was *AhTc1*, and the high‐level expression of *AhTc1* confers to the phenotype of purple testa in peanut.

### Identification of differentially expressed genes (DEGs) between pink and purple testa cultivars

**Figure 5:**
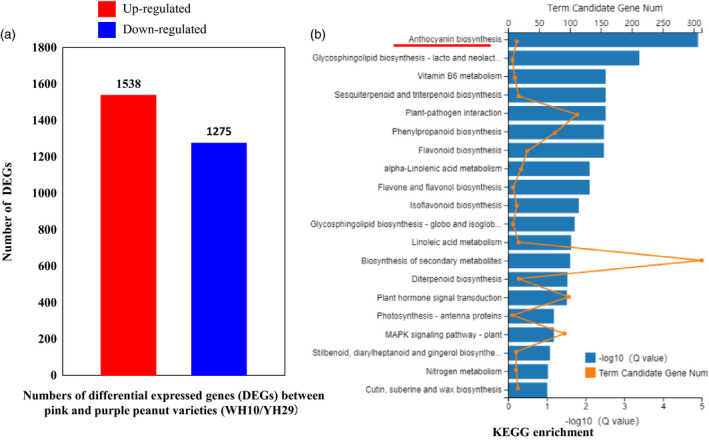
RNA‐seq analysis between WH10 and YH29. (a) Number of differentially expressed genes (DEGs). (b) KEGG enrichment analysis.

To further reveal the roles of *AhTc1* and gene expression regulation in testa colour of peanut, we employed RNA‐seq technology to analyse the genome‐wide gene expression profiles for the pink (WH10) and purple (YH29) testa cultivars. RNA‐seq generated a total of 64.97 and 65.14 Gb clean reads from WH10 and YH29, and 82.43% and 82.90% of them could be mapped with the genome of Tifrunner, respectively. Between the WH10 and YH29, a total of 2814 DEGs were identified under the criterion of log_2_Ratio ≥ 1 (Figure 5a). Compared with WH10, the expression level of 1538 genes were up‐regulated, and 1275 genes were down‐regulated in YH29 (Figure 5a). In order to get a better understanding of the transcriptome differences between two peanut varieties, KEGG (Kyoto Encyclopedia of Genes and Genomes) analysis was performed. We found that the many pathways were significantly enriched including ‘anthocyanin biosynthesis’, an important pathway that might contribute to regulating the synthesis of pigment and testa colour (Figure 5b).

## Discussion

### *AhTc1*, encoding a MYB transcript factor, regulated purple testa in peanut

In this study, the evidences of forward genetics and reverse genetics all supported that *AhTc1* was the key gene in controlling purple testa in peanut. Functional annotation and sequencing alignment showed that *AhTc1* encodes a member of R2R3‐MYB transcript factor. R2R3‐MYB represents one of the largest transcript factor gene families in plants. R2R3‐MYB transcription factors were involved plant development, metabolism and responses to biotic and abiotic stresses (Carre and Kim, 2002; Chen *et al*., 2017; Dubos *et al*., 2010). Previous studies showed that a distinct clade of R2R3‐MYB transcription factors played key regulatory roles in anthocyanin biosynthesis (Chen *et al*., 2017; Lin‐Wang *et al*., 2010; Liu *et al*., 2015; Nguyen and Lee, 2016). Anthocyanins, belong to the flavonoid family, are one of the most important pigments which can increase red, blue and purple colours in a range of flowers, fruits, foliage, seeds and roots (Lin‐Wang *et al*., 2010; Tanaka *et al*., 2008). In plant, the biosynthesis of anthocyanin was regulated through a complex MYB, basic helix‐loop‐helix (bHLH) and WD‐repeat proteins (MYB‐bHLH‐WD40, MBW) complex (Baudry *et al*., 2004). In the complex, the activity of R2R3‐MYB might determine the patterning and spatial localization of anthocyanins, different family members of R2R3‐MYB contributing to the accumulation of anthocyanin in tissue or developmental specific patterns. For instance, in apple, *MdMYB1*,* MdMYBA* and *MdMYB3* regulate the synthesis of red pigmented anthocyanins in the peel, *MdMYB10* regulates the synthesis of anthocyanins in the peel, flesh and foliage, and *MdMYB110a* controls the accumulation of red in the fruit cortex during the later phase of fruit maturity (Chagne *et al*., 2013; Espley *et al*., 2007; Liu *et al*., 2015; Takos *et al*., 2006; Vimolmangkang *et al*., 2013). Despite the important roles of R2R3‐MYB and MBW complex in regulation of anthocyanins synthesis have been well elucidated in many other plants, however, functional information of R2R3‐MYB in peanut is scarce, especially in regulation of testa colour of peanut.

### Different KEGG pathways involved testa colour in peanut

In this study, we identified a total of 2814 DEGs between the pink and purple testa colour peanut varieties (Figure 5a). Through KEGG analysis, we found that many of the pathways related to synthesis of other metabolites were also enriched, such as ‘vitamin B6 metabolism’, ‘Sesquiterpenoid and triterpenoid biosynthesis’, ‘phenylpropanoid biosynthesis’, ‘flavonoid biosynthesis’, ‘flavone and flavanol biosynthesis’ and ‘isoflavonoid biosynthesis’, these results implied that there might be the differences in flavour and other nutritional ingredient between purple and pink peanut varieties. Moreover, ‘plant–pathogen interaction’ was also identified as enriched between two peanut varieties (Figure 5b). Past studies showed that the accumulation and localization of anthocyanins and flavonoids were involved in stress resistance (Chalker‐Scott, 1999; Treutter, 2005). For example, in barley, the content of proanthocyanidins in testa layer was involved in defence against *Fusarium species* (Skadhauge *et al*., 1997). However, there is no evidence currently to show the resistance different between the purple and pink testa peanut varieties. In addition, GO (Gene Ontology) analysis also showed that many stress‐related GO terms were enriched, such as ‘response to wounding’, ‘regulation of jasmonic acid mediated signalling pathway’ and ‘regulation of defence response’, which supported the results of KEGG analysis (Table S4).

### BSA, BSR and QTL‐seq technologies for QTLs/genes identification

BSA was first reported in mapping the disease resistance genes in lettuce (Michelmore *et al*., 1991). During the past decades, BSA has been used to map the quantitative trait locus (QTL) controlled by single gene or major gene. BSA can be used for mapping the traits, which is opposite or with significant differences between parents, and having enough individuals with an extreme phenotype in the segregation populations. BSA can quickly figure out the molecular markers closely linked to the target gene (trait) without genetic linkage map (Poulsen *et al*., 1995; Salunkhe *et al*., 2011; Yuan *et al*., 2013). Recently, a series of NGS‐based BSA approaches were developed, including MutMup, MutMup+, BSR‐seq and QTL‐seq (Abe *et al*., 2012; Fekih *et al*., 2013; Steuernagel *et al*., 2016; Takagi *et al*., 2013). QTL‐seq is a whole‐genome resequencing (WGRS)‐based approach and has strong advantages in the species with reference genome (Takagi *et al*., 2013; Zhang *et al*., 2018). In this study, *AhTc1* was mapped using QTL‐seq in peanut. BSR was used to verify the QTL‐seq result. QTL‐seq and BSR had the same result, suggested that BSR was also an effective method for primary mapping of QTLs. In comparison with BSR, QTL‐seq could generate more polymorphisms SNPs, randomly distributed in the genome, which is important for narrowing the candidate regions. For BSR, more SNPs could be generated from the highly expressed genes. So, many SNPs in the noncoding sequences and noncoding region of genes could not be indexed. The cost of BSR was lower than that of QTL‐seq. QTL‐seq and BSR will be very useful in further peanut QTL mapping studies.

### Applications of *AhTc1* gene in peanut breeding

Testa colour is an important trait for peanut breeding programme. In addition to appearance quality, the purple testa peanuts contain higher levels of anthocyanin and many other microelements than pink testa peanuts (Attree *et al*., 2015; Kuang *et al*., 2017). The unknown genetic and molecular mechanisms regulating testa colour limited the molecular breeding of purple testa peanut. Several attempts have been made to analyse the genetics of purple testa formation and identify the potential markers for purple testa (Branch, 1985, 2001). However, most of the studies failed to provide tightly linked markers and useful information on the candidate genes controlling the purple testa. Since the testa colour is a maternal gene determined trait, the segregation occurs in the following next generation. The traditional breeding faces more difficulties for purple testa peanut selecting and breeding. In this study, the purple testa controlling gene *AhTc1* was firstly mapped in 4.7 Mb region of chromosome A10, and a series of closely linked markers were developed (Table 3). The utility of these markers will accelerate the process of purple testa peanut breeding. In addition, we identified the candidate gene, *AhTc1,* a R2R3‐MYB transcription factor coding gene. The expression level of this gene was related to the colour of the plants, providing important target gene for testa colour modification using transgenic or gene editing methods.

## Conclusion

The major gene regulating purple testa of peanut, *AhTC1*, was mapped in a 4.7 Mb region in chromosome A10 using QTL‐seq approach. A series of SNP markers were developed and genotyped for narrowing the candidate region, and a R2R3‐MYB transcription factor gene was identified. The evidences of forward genetics and reverse genetics all supported that R2R3‐MYB transcription factor gene was *AhTc1*, conferring purple testa phenotype. This work lays the foundation for the further understanding of the regulation mechanisms of peanut purple testa formation and molecular breeding of new varieties with purple testa.

## Materials and methods

### Plant materials and anthocyanin content determination

Weihua 10 (WH10), Zhonghua 8 (ZH8) and Yueyou 20 (GT‐C20) are peanut varieties with pink testa. YH29 and Zhonghua 9 (ZH9) are peanut varieties with purple testa. WH10 and GT‐C20 with pink testa were crossed with the purple testa variety YH29, respectively. In addition, ZH8 with pink testa was crossed with a purple testa peanut variety ZH9 to produce F_7_ recombinant inbred lines (RILs). All F_1_ hybrids were evaluated using SSR and MITE transposon markers (Qiu *et al*., 2018). Anthocyanin content of peanut testa was determined using the method reported in previous studies (Mancinelli *et al*., 1991; Serrano *et al*., 2012).

### Nucleic acid isolation and bulked libraries construction

Peanuts were planted in the field of Shandong Academy of Agricultural Science (SAAS) in Jiyang. To prepare the bulk DNA, leaves deriving from 30 pink testa and 30 purple testa F_2_ lines were collected for DNA extraction, respectively. Total DNA was extracted using Plant Genomic DNA Extraction Kit (TIANGEN, Beijing, China) according to the instructions of the manufacturer (http://www.tiangen.com/en/?productShow/t1/4/id/10.html). Then, pink‐pool and purple‐pool were constructed by mixing the equal amount of DNA from 25 pink and 25 purple F_2_ individuals, respectively. For constructing the BSR‐seq libraries, leaflet (unfolding young leaves) from 30 purple and 30 green F_2_ individual lines of KF1 population, 10 purple and 10 green F_2_ individual lines of KF2 population, and 19 purple and 19 green F7 individual lines of ZH population were collected, respectively. RNA was extracted using RNAiso Plus (Takara, Dalian, China) according to the instructions of the manufacturer (http://www.takarabiomed.com.cn). The pink‐pool and purple‐pool for each population were constructed by mixing the equal amount of total RNAs. The integrity and purity of the DNA and RNA samples were determined by 1% agarose gel electrophoresis, and the concentration of DNA and RNA was measured in NanoDrop. High‐throughput sequencing for RNA and DNA libraries was performed by Novogene (http://www.novogene.com/) using an Illumina HiSeq^™^ 2500 system with 150 bp*2 paired‐end sequencing.

### QTL‐seq, BSR‐seq and bioinformatic analysis

To obtain clean reads, the low‐quality reads (*Q *≤* *20), adapter sequences, *N *>* *10% reads and too short reads (<20 bp after trimming the adapters) were removed by tool Trimmomatic. Then, all clean reads were mapped with the genome sequences of *Arachis hypogaea* (PeanutBase version 1.0, https://peanutbase.org/) using mem module of BWA software with the harder filters of minimum number of identical bases 40 (Li and Durbin, 2009) for QTL‐seq data, or using mapper STAR for BSR‐seq data. The alignments were further filtered with criteria that require minimum 50 bp identical bases to the reference sequence, maximum 5% mismatch rate, maximum 5% clipped ends, maximum 50 bp gap, maximum 2 alleles and minimum 50 mapping score. The germline SNP was called using HaplotypeCaller module of GATK tools software following the best practice protocol. The raw SNPs/Indels were further filtered using requirements that only two genotypes exist, total sequencing depth >20 and <10 000, mutant allele sequencing depth >5, proportion of either allele >5%. The allele frequency was calculated based on the filtered read counts (discarded inner low‐quality genotypes) of both alleles at the SNP/Indel sites. Then, AFD (allele frequency difference) was the absolute value after subtraction of the allele frequency of the purple‐pool from that of the pink‐pool. Fisher exact test was applied to evaluate the allele frequency difference as well using read counts. If *P*‐value < 1e−5 and AFD > 0.5, the SNP was regarded as candidates linked to the trait.

### STS markers development and candidate SNP validation

To develop the markers for the validation of QTL‐seq results and narrow the candidate region, the sequences containing 1500 bp upstream and 1500 bp downstream of SNPs were downloaded from PeanutBase (https://peanutbase.org). Due to the high similarity of A and B subgenomes, it is difficult to design the primers for only amplifying the A or B allele. Thus, the 3001 bp sequences were used to blast with the genome sequences of cultivated species Tifrunner, donor ancestor species *A. duranensis* (A genome) and *A. ipaensis* (B genome). According to the alignment results, we designed the specific primers only for A using primer premier 5.0 software (primer premier 5.0 software). PCR product direct sequencing was used for genotyping of these STS markers.

### Gene cloning, vector construction and gene transformation

The ORF region of *AhTc1* was amplified using EX Taq HS (Takara, Dalian, China) from ZH9 and cloned into pMD^™^19‐T Vector (Takara) according to the instruction of manufacturer. The following primers were used: TestaORF‐F (5′‐TGCTCTAGAATGGAGGGATCCATAGGCCT‐3′) and TestaORF‐R: 5′‐AACTGCAGTTATTGTGGATCCCACAAAT‐3), in which the unique *Xba* I and *Pst* I sites were introduced at the 5′ and 3′ ends of the ORF of *AhTc1*, respectively. The plant expression vector containing 35S:*AhTc1* was constructed by cleaving the ORF was from T vector and recombining into pCAMBIA2300‐35S‐OCS vector, and using CaMV 35S promoter to direct the expression of *AhTc1*. The insertion of the *AhTc1* construct was confirmed by PCR as well as enzymatic activity assays. The recombined vector was transferred into *Agrobacterium tumefaciens* LBA4404 and then used to transform *Nicotiana tabacum* cv Nc89 using leaf disc method (Horsch *et al*., 1986). Transformed seeds were selected for kanamycin resistance, and the transgenic lines were confirmed by PCR method (Figure S2).

## Conflict of interest

The authors declare that they have no conflict of interest.

## Author contributions

XW and CZ conceived and designed the experiments. YZ, JM, ML, LD, GL, HX, SZ, LH, PL, MY, LR and JG performed the experiments. CZ, CM, PL and YZ analysed the data. CZ, XW, and BZ wrote and revised the manuscript.

## Supporting information


**Figure S1** Confirm the QTL‐seq results using BSR in three populations.

**Figure S2** Schematic map of the transgene construction. A: schematic diagram for construction of pCAMBIA2300‐35S‐OCS‐AhTc1. B: Transformation, regeneration and transplant. C: PCR detection of transgenic lines.

**Table S1** Detail information of SNPs identified using whole genome resequencing.

**Table S2** Gene information in the candidate region on chromosome A10.

**Table S3** Genomic and cDNA sequence information of candidate gene J3K16K.

**Table S4** Top 30 enriched GO terms between pink and purple testa colour peanut varieties.
