# Supplementary material for: Whole‐genome resequencing‐based QTL‐seq identified AhTc1 gene encoding a R2R3‐MYB transcription factor controlling peanut purple testa colour
Source: Plant Biotechnol J. 2019 Jun 12;18(1):96–105. doi: 10.1111/pbi.13175 (PMC6920131; doi:10.1111/pbi.13175)
Supplement: Supplementary file 1 — Figure S1 Confirm the QTL‐seq results using BSR in three populations. [file PBI-18-96-s005.pdf]

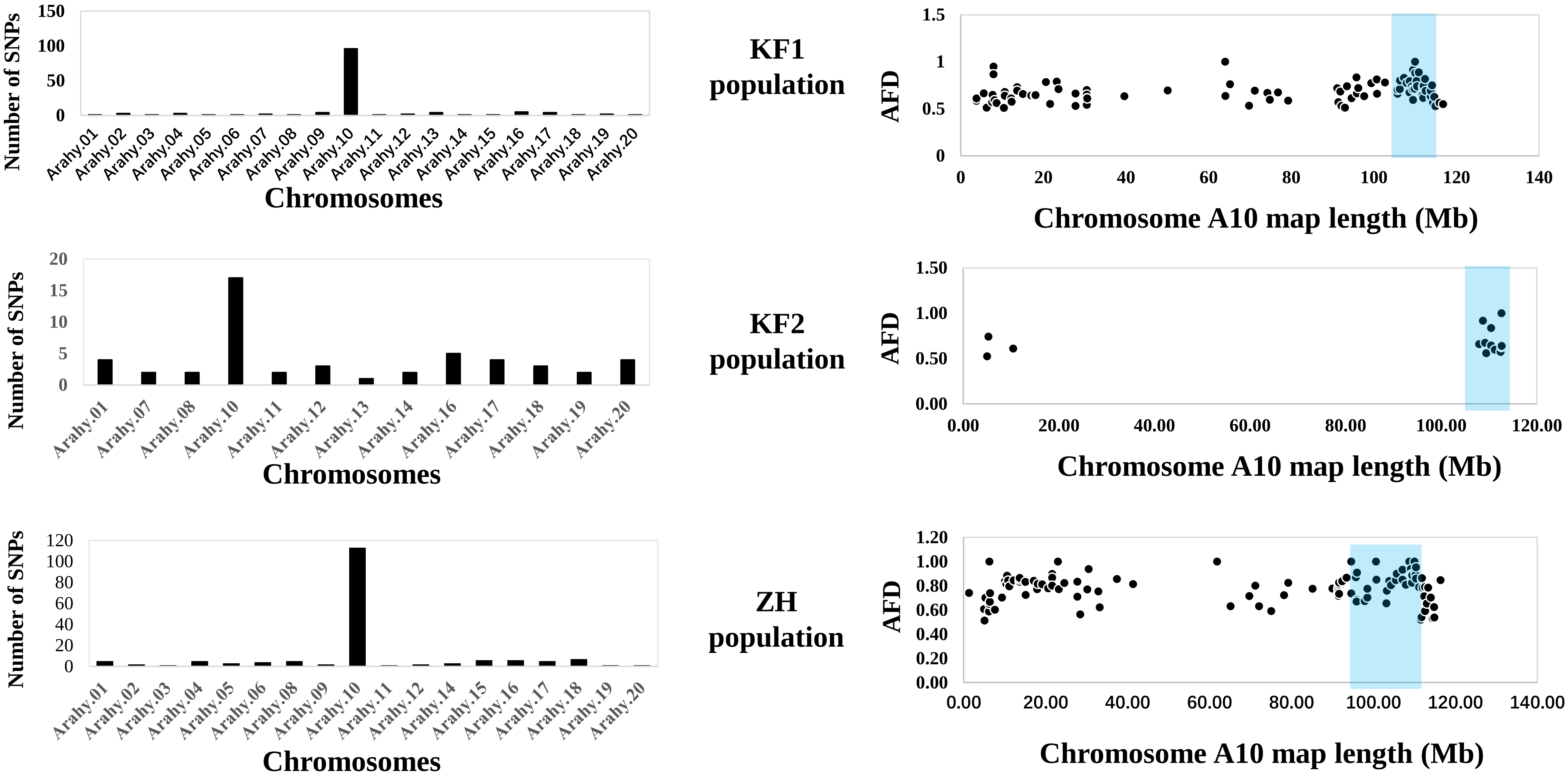

Figure S1 Confirm the QTL-seq results using BSR in three populations
