# Supplementary material for: Whole‐genome resequencing‐based QTL‐seq identified AhTc1 gene encoding a R2R3‐MYB transcription factor controlling peanut purple testa colour
Source: Plant Biotechnol J. 2019 Jun 12;18(1):96–105. doi: 10.1111/pbi.13175 (PMC6920131; doi:10.1111/pbi.13175)
Supplement: Supplementary file 2 — Figure S2 Schematic map of the transgene construction. A: schematic diagram for construction of pCAMBIA2300‐35S‐OCS‐AhTc1. B: Transformation, regeneration and transplant. C: PCR detection of transgenic lines. [file PBI-18-96-s004.pdf]

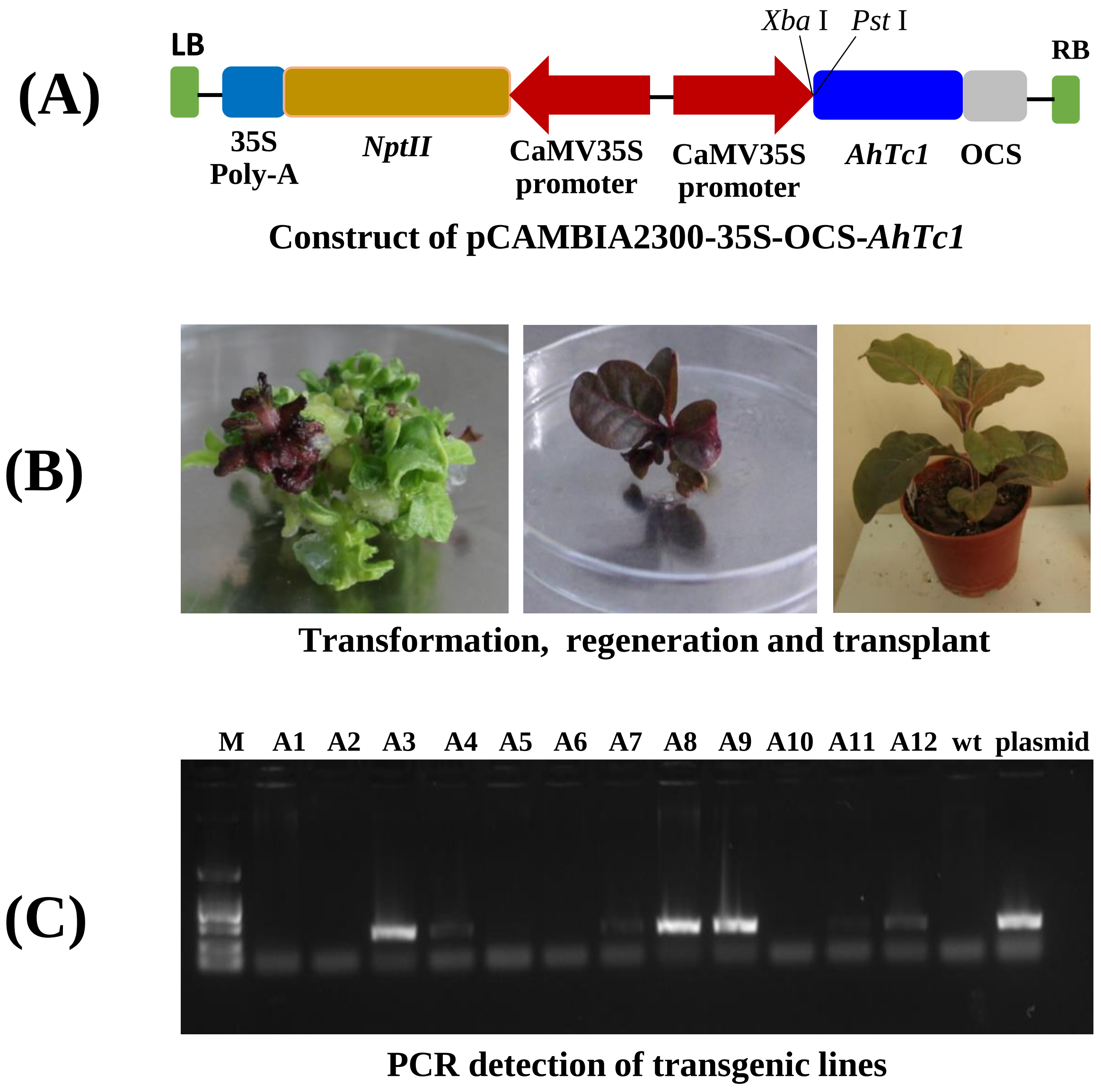

Supplemental Data Figure S2. Schematic map of the transgene construction. A: schematic diagram for construction of pCAMBIA2300-35S-OCS-*AhTc1*. B: Transformation, regeneration and transplant. C: PCR detection of transgenic lines
