# Supplementary material for: Whole‐genome resequencing‐based QTL‐seq identified AhTc1 gene encoding a R2R3‐MYB transcription factor controlling peanut purple testa colour
Source: Plant Biotechnol J. 2019 Jun 12;18(1):96–105. doi: 10.1111/pbi.13175 (PMC6920131; doi:10.1111/pbi.13175)
Supplement: Supplementary file 5 — Table S3 Genomic and cDNA sequence information of candidate gene J3K16K. [file PBI-18-96-s002.docx]

**Supplemental Data Table S3 Genomic and cDNA sequence information of candidate gene J3K16K**

| >genomic DNA sequence  TGTTTTTTTTTTCAATATTTGACAAATAATTTTTTTAAAAATACAAAAATAAATTAATCCTTACAAACTCTAAAAGCGCATGTAGCAAATTAATCTTTATTTCACTTTCTAAAAATAGTAGAATAGTAGAGGAATCAAATTGTTTAAAAGGATAGATAATTAACTCATTTTTATTTTTTGTTTTATTAAAATTAATTTTTTTAATATTATAAAAGAATTTAGGGACCAATATATCAAGAATCTAATTAGAAAATAATTTTTTCATATTAAAGTTGATCAGTGATCAATTTGAGCTACTACACTTATTTATTAGGATAAAGTACTATTTTAGTCCCAAACGTTCGGCCCAAATTCCAATTTGATGATCTCTAATATTTCAAATATCTTATTTCAATTTTAAAAAGTTTTAAATATATTCAATGTTGTCTTATCATTCGATTTGACTTGAATAATTAACATAAAAACACTACAAGACAAACAAATAATTGCGGCGGTTTATTTGTCGATTTGCGGCGATTTTGAACCGCCGTTAAACAAGACTCCAGTAGAACAATCACCGCAGTACATAAAGGCGGGGGATTGCATTTTGCGGTGGTTTCCAGCAACTGCTGGCATAACCGTCGAAAATCAGTAATTTTGCGTCGGTTAGATTAGCGGCGGTTTTATATTACGAAGAGAGGTTTCTTCCACGTATTTGCGACGCTCCTTAACCGCCGCAATTTGCTGCTGCGTTTTTATATAAAATTTATTTGCGGCTGTTTTAAACCGTTGCTATTTGTAGCTCCTATTTCTTTCTAAAAATACTATTTTAGTTTGTTTTCTTTTATTAACCTGGGGATATTTTTGGAGTATTTTTTATTTTAAAAATTTTATCGATCTTTTATAAAAGTCTAACTTGATGTCGAGAAGTCATGAATATTTAAAAGCGAAATAAACTAATATATTTATTTATTTTAATATCAATAGAGCATTTTAATAGTCACAAAAAAAGTAAAGTATTTGGCTAATAAAAAATGCTGCATAGTTAACTTAAACAAAACATATACTAAAAAATAAAATATCACATATATCTTAATTTTTATTTTCTACTATGTCTATCTAAAGAATTCATTCATGTAGCGATGTCTGATAGTAACTCTCCACCTTACTGTTGAGTCAGATAACTTAGCACTATTTTATCTAACCAAAATAATTCTCTATTTTGGCAGCTATTTCAGTGCACCCTGCCATCTTTAAAATTGCCTAGATAACCATTTTCAAAACACCAAGCCCTATATTGCAAGCCAGGGAAAAAATGTTTCTGACATAAACATATATAGCTAATTTATACAATTGATAATAGCTATGTGCCTTAAAAAAAACCAAAGAAAGAAAATAGTTGATGAAATCTATATGAACAATGAGAGTTATATATATATATACTAGAATGAAAATAAGCTTGAACAATAGCTTTAGGCCATTGAGCATGAACATCTTAAATGAATTCATCTATAATTGATAGATATATACTAAAAAAAATCTGATAAACATCTACTAAAAAAAATTAACTAATCAATAAATGAAAACTTTAAACAAGAAAATAAACATTTGACAAAAATATTTTACCCACAAAACAATTTTTAAGTAGTTCTTAATTGTTAATGGATCGATTTTACTTATTTACTGAAATTGAATGTTATATTGACTTTTATATATGTTAATGGTTCATGCTAAATTTAACCATAGAACAACATTAAATCTATTTAAAATTTTTTGTGATCGAAATAAAACATTTAAAACATTAAAAACTAAATTAGAATTTAATTCAAAAGTTGGAGACGAAAATAGTAGATTACCTATTCATTAAAGTGACAGAGGTGCTGCTTTTTGTGGTACGTGAAGCATCCACGAAGTCACTTTTGCATTTTGTGGACACCGAAATTGCACGTTGATGCTTTGAAATGTCGTGCCCATAGTACTATATATATAAGTTAATATTGTTGGGTACAATGGCCATAGAACTCAACACTATCAAGCCTAGTGTTTTATTTGGTTAGTGAAAGTACACTGTGTTGCATCACTGCAAATAATAATTGAAGTGTATATTGTTTCATTTCCATGGAGGGATCCATAGGCCTAAGAAAAGGTGCATGGGCTAAGGTGGAAGATGACCTTCTAAGAGCTTGTGTTGAACAATATGGAGAAGGAAAGTGGCACCTAGTTCCTTCTAGAGCGGGTAATATAATTTAATTATTTAATCTTCATCAATAATGATAAACGTCTTAAGCATATTTTTAAATTTGTAAGTGTACCGAAATTTTTTTTTAATTTCAATTTGTGTATATCACGAATGTCGATAGGATATATAAACTATATAAAAATTTTATTGATATTGATAAAAGGTAATAAGTCTCCTTCGAAGAACCCTAAGAAGAAAAAAAAATAGAAGACATGGACAGAAACAATGTGTCTAGAGACACTGAATTAGTGTATTTTGTATCCATCCTAACAGGAAAGACACGAAGACACTAATAAGGAACACAACTTATTTTTTATTTTTTCTTTTATTATTCTTGTTAATTTTTCATAATTATATTTTTTATTATTATATTTTTTATCTCAAATTTTTTGAATGAAAAAAAATAAAAATAAATTGAATTTTCATAATTTGTTCTAGTTTATCACCAAACAGAATACAAGAACACAAAATTTTGTGTCTCTGTCCATTAGTGTCTTGTTCTGTCCTGTTCTCAGTGTCTTGTCCTATCCTGTTCTTAGAAACAAATGTAGCCTAATATATATCTACTACTCTCAAAAATTTAAACTGATAAAAATAGGCATATAAATAGTTATATATGTAATACGCATCTTCAAATAAGAATTTCTTTAAATTTATTTAAATTTTTGTATAGAATAAAGATCAAACTTTAAAAATTTTGATAATAATATAAAAATTTAAATTAATAAGAACATAAAACAAATAAATAGTTATATATCCAACATGAGAAAGTTTAGGTGGCCAGCATTTTTATTAAAATTTGGCCAACACTTAATCAACAAAAAAAAGTGAATAATCCTACACTATTAGATGTAATTTCACACTATTAAAAATACTGATAATAATTAATTGATAACTATAAATCACAAAATATGTTGGCCTAGCACTCCTCTAATACAAAATAAATATAAGCACATGTTCTAAAACTTGGGAACTAGAAAACGCCGATCACTTTGAAAACTTGCTCAAGATAAAGCTTCTAGTATACTAATCTAAAAATATACCGGGGAATAATAAGTTTTTGGATGAATATGATAATTAAAAATGAAAGAGTAAATTATCATGCAAGACATACATAAACACATTGAAACGTTAAAATGCTTAGCAAATCCATATTGATGCAGTGATGATGAACCATGAAAAGAGCTTAGCAAGAAGAAAAGAAAAATATATTTTAAAATATTATTGAATGTTCATATATTTGCTAATTATTTCTCTACATATGACTTAATTCAGATATACAAGTAACGAATTATATATATCGAGATAGTGTTAGTTGGCATATATTAATAACAGTTATTGTTAAATTTGTGCATGGATGACAGGGTTGAACAGATGCCGCAAAAGTTGTAGACTGAGATGGTTGAATTATCTGAAACCAAATATAAAGCGTGGAGAGTTCTCTGAAGATGAAGTTGATCTCATGATTAGAATGCACAGACTTTTGGGAAACAGGTGGGTTCACAACCATAAATTCAACTATATATAATATATTATTTATGGTAAAAACTTAGGTGCAGTCGATTTCACATGAAGTTGATAACTGAGAGCTGTTAGATGGTTTAATTGATTTGACTAAATTTTCATTTAACGATTCTCAGCTATCAACTTCACATTAAATTCACTGTATTTGAGTTTCCACCATTATTTATACAATGATATTTTCATATATTTAATTAAATTATTTAATATAATAAAAATCTATTATTTTCACATAAAAATACTTTAAAATTTCAATAGTCTACTAATAAAACTATTATGACTAAGTAATGATTTTAACAATTATATTATATGTATATAAAAAATTAGTTACAAAATTAGTTATTTATATAAAATATATATTAAAATATAAAATATATATTAAAAATAATTAAATATATAATAGTTGGTTTTTATGTAAAGATAAATATTATATTTTTTATTTTAATGATTTGGATTCAAATGCAATACTCTTTTAGTCCGTAGATACGCATGTGGGAATCAATCCTATTTTCTATACAAACATTTTCTGTTATCCTAATTAAAAGAAAGTAGATATTTTTTTATAAAATAATATTATAAATCATTAAATAGTTTCATATGTTTAATTAAATATATTTAATTAATCATTTAAAATTTACAGTGTCATCTTCATAAAAAATATAATTATATAAATATTTTTATGTGAGAAAACACATAACATAGAACTATATTGGTAACTTATTCTTTTTCAACACATCTCTTCTCTTCTTCTTCTTTACTTCCTCCACCTTAAAGCCATTCCTCCATGCATGACTGTATACAAAATTTTGGTTGTCATACTTTTATTATTGGAATACATATCTTCCTATATATAAATATATAATTATATTAGATATACATTAGTCATTAATATAAAATATATATTAAAATATAAAATATATATTAAAAATAAATTAAATTATATATATTTATATATAAATATATAATAACTAAATTTAATGTACAGATAGTATTTTTAAAAAAGATATAATGAAATAACAACCATAAAACTAGTTACAACTTACAAGAAATATAAGATCTACTGAGATGATATAATGATTTATAAGCCACTCATACCGGCAGCGGTGATGCCTAATAAATTGGTTAAAAAATAAATTAATGGACATATTATGCCCTAATCCTAGACACGCTTATTAATCTAATTTTTCATTTATATTCTAAAGCAAAATTATTTAATAATAAAAGGTATAGTAAATAGAAAAGGAAAAAAGCACAGACTCTTTATTTTATGTTATAAATAAAAAAAATATTTTTAAAAATATCTAAAATAAATCTTTCTGAAATTTACTTGTATTTATCAAAAGAGTAATGATAAAAAAGATAATAAATCTAAAATTATTTTATTTATTATGTATATAATATTTAAAATTATATATTTATTGCATCTAACATTATGTGTTTATTTCAGAAATATTTAATAAATATAAAATAAAATAATTTTGAACTGATTTTTATTATCTCTCAAATATTTTTGTTATTAAAATTAAAATATCTAATATAAGTTCATACATTAATAGTTTTCCAAATTTACTATAATATTTATATTTATTATAATTTTTAAAATTTTAAAAATTATTTTACTAAACAAAATTATTATTACTTTTAAAAGAATAAACTAATTTCAAAAAATAAAAATTTTATCAAACCAAACCTAACTCTAATAAGTAATAAGAAAGTCCTGTAAGTGTGTAGTAACATACTCATAATAATTCTTGAGATAATATAATACAACTAATTATAACCTTTTTATAATTAAATTTTAATAATACAATTACAACCTTTTAGCTTATCATTTAATTAATTTTTTTAAAAGACTCTTCTAGTAATGGACATGAAAAATAGGTTTTTTTTTTCTTTTCATCTATATGATTTTGTTAAGGAATCAACATTTTTTAAATTAATAATTAGCTAATAAAATAATTGACGAATAAAATTAATTAAAATTAAAAAATAGTTTATTGTTGAAGAAAAAAACTGAGATATGTTTGATTTGTGTTTTTCAATTTTATTTTTATTCAATATTTTTAATGTGTACAATTTTATAAAAGAAAAAATAAAATAAAAAATTTATTATCCTTATTTTTTTTAACCAAAATTTTAAAAATTAAAAACACAATGTACACGTGATTCATCACTTATACCATTTTTTGGATGACAAGACTCATATGAAATTAATAAATAATTTAATAAATTTAATTAAATTATGGTCTAACGGTTTTTAACTATGAATTTTATATACAACTGCATTGAAGTTTTCACCTTTCTGTTATTATTGTTTTGCGCGTAGCTAAACGTGAAGTTCATCTTCATCTTCTGGTTTTAGTTTGACTAAGCAATGGTAGTTTATTTATGCACTGATGGATTAATATGAATAACAACATGTAGATGGTCCTTAATTGCTGGAAGACTTCCGGGAAGAACGCCAAACGATGTGAAGAATTACTGGAACACCTATGTTCGAAGGAATAATAAGCACACTTCATCATCATCATCAGTACCCTCTCCTTCATCAGTGGTGACGACAGTGAAACGTCATGACCATATTAATCATCAGGTAATAAAACCTCATCCCCGAACTTTCTCGAAAGCATCGCCATGGTTATTATTAAAGAAAACATCTACAAGTGGTTATCATAATAATAACAAGCAGAGGGATGGGGCCAAGGCAGAAGAAGAGTGCACCGATAATAATAATAATAATAATGATAATAATGGTTCTGGTGGTGGATGTGATGATGGGAACACGTGTCGGAACAAGAACGGTGGCAAAGGGGATGAAGAGAAGTTGAACGACGGGTGCTTACTCTCAGTCTCAGGTGCTGAGGAAGAAGAAGAAGAAGAAGACTGGAAGCTGCTTTTACCTGACTTCAATTGGGATGCTCATAATGATGATCACTTCTTGAAGGACGCTTCTGATGCTGACCACGATATGCTTATTAATGTCCATGGTCAAACTTGGAGTGATATCCTTCTTGATATCAATTTGTGGGATCCACAATAATTTTGTTTTTTTAATTGCTAATGAATTATTGCATGTATAACGATTGATAATTATTCTGATAATTATTTAAGACGACGAATAATTATTTGCTAATATAAGTTGGTTATTTGAGTAGTCATCTATATTCTGCTGAATTAAAGCTTCCTTCATGATCCTAGCGGAGATGTTCGAAACAATCTGGTGCTGTTTGGGAGCTTTTGAACTCTTTATCGACTTCATGGACTCTGTCCCATCAGGGGCATATGGCTCCGGATTGAGAATCCTTTCCTACCTCTTCCCGATTGATGAAGCTCCTTATCCACCAAAACTGAAATAAAAAATATATATATATATATAGTTATAAAATTTTTGATAATATTAAAAAAACAAAAAAACCTAAAAATAATTTTATTTAATATTTATTAATTATCATAATAATTAATAAATATTAAATAAAACAAATTAAAATTATTTTTGGCTGATTTTTTTTTTACCAAATATTTTCCCTTTACTTTACACAATGATTAACAATCTTTAGGCTTATCAAC |
| --- |
| >ORF sequence  ATGGAGGGATCCATAGGCCTAAGAAAAGGTGCATGGGCTAAGGTGGAAGATGACCTTCTAAGAGCTTGTGTTGAACAATATGGAGAAGGAAAGTGGCACCTAGTTCCTTCTAGAGCGGGGTTGAACAGATGCCGCAAAAGTTGTAGACTGAGATGGTTGAATTATCTGAAACCAAATATAAAGCGTGGAGAGTTCTCTGAAGATGAAGTTGATCTCATGATTAGAATGCACAGACTTTTGGGAAACAGATGGTCCTTAATTGCTGGAAGACTTCCGGGAAGAACGCCAAACGATGTGAAGAATTACTGGAACACCTATGTTCGAAGGAATAATAAGCACACTTCATCATCATCATCAGTACCCTCTCCTTCATCAGTGGTGACGACAGTGAAACGTCATGACCATATTAATCATCAGGTAATAAAACCTCATCCCCGAACTTTCTCGAAAGCATCGCCATGGTTATTATTAAAGAAAACATCTACAAGTGGTTATCATAATAATAACAAGCAGAGGGATGGGGCCAAGGCAGAAGAAGAGTGCACCGATAATAATAATAATAATAATGATAATAATGGTTCTGGTGGTGGATGTGATGATGGGAACACGTGTCGGAACAAGAACGGTGGCAAAGGGGATGAAGAGAAGTTGAACGACGGGTGCTTACTCTCAGTCTCAGGTGCTGAGGAAGAAGAAGAAGAAGAAGACTGGAAGCTGCTTTTACCTGACTTCAATTGGGATGCTCATAATGATGATCACTTCTTGAAGGACGCTTCTGATGCTGACCACGATATGCTTATTAATGTCCATGGTCAAACTTGGAGTGATATCCTTCTTGATATCAATTTGTGGGATCCACAATAA |
